# Capacity of National Malaria Control Programmes to implement vector surveillance: a global analysis

**DOI:** 10.1186/s12936-020-03493-1

**Published:** 2020-11-23

**Authors:** Tanya L. Russell, Robert Farlow, Myo Min, Effie Espino, Abraham Mnzava, Thomas R. Burkot

**Affiliations:** 1 Australian Institute of Tropical Health and Medicine, James Cook University, Cairns, Australia; 2 R. Farlow Consulting LLC, Burkeville, TX USA; 3 Asia-Pacific Malaria Elimination Network, Singapore, Singapore; 4 African Leaders’ Malaria Alliance, Dar es Salaam, Tanzania

**Keywords:** Malaria, Anopheles, Vector surveillance, Capacity building, Needs Assessment, Logic framework

## Abstract

**Background:**

Solving the problem of malaria requires a highly skilled workforce with robust infrastructure, financial backing and sound programme management coordinated by a strategic plan. Here, the capacity of National Malaria Control Programmes (NMCPs) was analysed to identify the strengths and weaknesses underpinning the implementation of vector surveillance and control activities by the core elements of programme capacity, being strategic frameworks, financing, human resources, logistics and infrastructure, and information systems.

**Results:**

Across nearly every country surveyed, the vector surveillance programmes were hampered by a lack of capacity and capability. Only 8% of NMCPs reported having sufficient capacity to implement vector surveillance. In contrast, 57%, 56% and 28% of NMCPs had the capacity to implement long-lasting insecticidal nets (LLINs), indoor residual spraying (IRS) and larval source management (LSM) activities, respectively. Largely underlying this was a lack of up-to-date strategic plans that prioritize vector surveillance and include frameworks for decision-making and action.

**Conclusions:**

Strategic planning and a lack of well-trained entomologists heavily hamper vector surveillance. Countries on the path to elimination generally had more operational/field staff compared to countries at the stage of control, and also were more likely to have an established system for staff training and capacity building. It is unlikely that controlling countries will make significant progress unless huge investments also go towards increasing the number and capacity of programmatic staff.

## Background

The World Health Organization (WHO) recommends both malaria control and prevention strategies [1–5]. The evidence-based core strategies include vector control with long-lasting insecticide-treated nets (LLINs), indoor residual spraying (IRS) and the supplementary strategy of larval source management (LSM); along with access to diagnostic facilities and improved treatment as well as surveillance, monitoring and evaluation (including entomological surveillance). Wide scale deployment of these strategies reduced the global malaria incidence by 37% for all human malarias [6]. Furthermore, between 2007 and 2019, 11 new countries were certified as malaria free—the first certifications since 1987 [7, 8]. However, the global progress has stagnated since 2014 [9]. Predicted trends in climate change and urbanization will likely facilitate reductions in malaria transmission, but will not, by themselves, achieve elimination [10]. These megatrends facilitating malaria control will be offset by disruptions to health systems though civil unrest, migration of displaced populations and land use changes. The capacity of National Malaria Control Programmes (NMCPs) will be further challenged by public health emergencies, such as the COVID-19 pandemic [11]; although it is important to note here that most countries have been supported to develop and implement mitigation plans to prevent the potential negative impact of COVID-19.

Additional biological challenges are escalating, most notably in the form of physiological [12, 13] and behavioural [14, 15] resistance of mosquitoes to insecticides and resistance of parasites to anti-malarial drugs [16]. With the growing number of pre-qualified malaria control products available and recommended for programmatic deployment, malaria control has evolved into “a problem to be solved, not simply a task to be performed” [17] with country programmes encouraged to adapt WHO recommendations to local circumstances.

Understanding the level of capacity of National Malaria Control Programmes to implement vector surveillance and control is essential to identify bottlenecks and needs that can be supported with capacity building. Solving the problem of malaria requires a high level political commitment together with a skilled workforce, supporting infrastructure, financial backing and sound programme management coordinated by a strategic plan [2, 4]. Of note, the current WHO guidance highlights the need to stratify transmission scenarios using local data on vector distributions and their associated behaviours, including insecticide resistance [18], alongside monitoring of intervention access and use as well as their impacts on vectors and transmission to proactively manage the challenges that will inevitably arise [1]. Thus, effective control will increasingly depend on surveillance of both malaria cases and vectors as a core intervention [4]. However, less than half of the WHO minimum recommended vector indicators are monitored annually by countries and significant gaps exist between the data collected and the actual use of data in making programmatic decisions [19, 20]. The limited and shrinking cadre of vector control officers (at both the technical and managerial levels) are hypothesized to be major threats to malaria control programme effectiveness [21] along with insufficient finances and infrastructure [22], but the scope and scale of these limitations is unverified.

The objective of this manuscript was to define the level of existing capacity of NMCPs to implement core vector surveillance and control activities. Conducting vector control needs assessments was defined as a key priority in the Global Vector Control Response (GVCR) [2]. The needs assessment was implemented using an online rapid assessment tool, and the results were analysed using a logic framework delineating the core elements of programme capacity, being strategic frameworks, financing, human resources, logistics and infrastructure, and information systems.

## Methods

### Data collection

As malaria surveillance, including vector surveillance, has been defined as a core intervention [4], the term “vector control interventions” as used here includes vector surveillance. Information on the capacity of NMCPs to deliver vector interventions in endemic countries and a country that recently eliminated malaria was collected using an online survey instrument (available in English, French and Spanish at https://ee.kobotoolbox.org/x/#YNbC) [20]. The survey instrument was designed in consultation with the Asian Pacific Malaria Elimination Network, the African Leaders Malaria Alliance, the E8 Secretariat, the Malaria Consortium and the University of California-San Francisco Malaria Elimination Initiative. The directors and key personnel of National Malaria Control Programmes and their technical partner organizations participated in the survey. An initial manuscript [20] analysed and presented the malaria vector control and surveillance services delivered, including vector control interventions in use, how intervention access and use is monitored, the vector surveillance indicators monitored, technical methods to quantify vector parameters and how data was managed and used in decision-making. Here, the strengths and limitations of the capacity of NMCPs to implement core vector control and surveillance activities at the national and subnational (provincial and district) levels was analysed.

### Statistical analysis

**Fig. 1:**
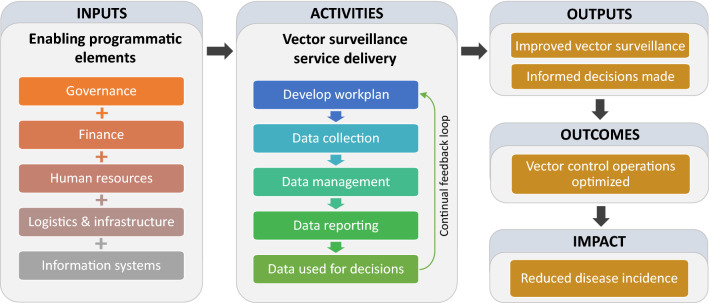
Framework for vector surveillance which can be used as a basis for conducting a needs assessment of the inputs and activities of vector surveillance programmes

Functional malaria programmes are built on core programmatic inputs: governance (strategic plans and guidelines), finance, human resources, logistics and infrastructure, and information systems (Table 1, Fig. 1). The programme logic model or framework (Fig. 1) delineates the standard against which the capacity and impact of programmes was assessed. Despite the linear presentation of the framework, vector control programmes are inherently complex with multiple feedback loops. Responses to any open-ended questions on capacity were coded against these core programmatic inputs, with more detailed analyses of 13 sub-categories (as defined in Table 1) [23]. This process was conducted independently by two of the authors (TLR and TRB) with discrepancies resolved by the third author (RF).

**Table 1:** Categories defining the capacity requirements for vector surveillance and control programmes

Major categories	Subcategories		Definitions
Governance	Higher level planning within the NMCP for entomological surveillance		
	Strategic plan		Document describing and outlining the full complement of recommended vector surveillance and/or control activities
Finance	Guidelines		Country specific standard operating procedures
	Money allocated for vector surveillance activities		
	Budget		Finances supporting the strategic plan
	Financial Management		System for disbursing funds
Human resources	Workforce engaged to implement the strategic plan		
	Professional staff		Planning and programme management personnel
	Operational staff		Personnel implementing the surveillance plan
	Training		Staff equipped with correct skills/knowledge
Logistics and infrastructure	The ability of the vector control programme to perform functions, solve problems and achieve objectives at an institutional level		
	Logistics system		The organization of programmatic functions
	Transport		The system or means of conveying people or goods from place to place (vehicles)
	Infrastructure		Physical structures and facilities (offices, laboratories and insectaries)
	Equipment and supplies		Consumables, equipment, traps and insecticides
	Supply chain		The sequence of processes involved in the procurement and distribution of a commodity
Information systems	The techniques and methods by data is collected, recorded and distributed		
	Data collection		Methods and forms used to record data
	Data management		Systems to record and analyse information
	Data communication		Systems to disseminate and present information

Two-sided proportional comparisons of the capacity of NMCPs in countries eliminating compared with those controlling malaria were analysed with a chi-squared test of proportions (*prop.test*). Questions with multi-categorical answers were compared between eliminating and controlling countries using a chi-squared contingency table (*chisq.test*) (e.g., subcategories of capacity limitations). The numbers of vector control and surveillance staff provided by the respondents was standardized against the size of the population at risk of malaria in 2018 [9]. The difference in the numbers of employees between controlling countries and eliminating countries was analysed with a generalized linear model (GLM; package *MASS*) with a Gamma distribution. The difference in the composition of national, subnational, field and laboratory staff between control and eliminating countries was analysed by permutational multivariate ANOVA (PERMANOVA; package *vegan*) [24]. These analyses were performed using the R package (v3.5.1). All country specific results are reported anonymously with results summarized by transmission status (controlling or eliminating malaria).

## Results

### Capacity for vector intervention deployment (includes surveillance and control)

Of the 35 participating countries (in Africa (n = 18), Asia–Pacific (n = 14) and the Americas (n = 3)), seven were classified as “eliminating” based on their inclusion in the E2020 (n = 6) with one (Sri Lanka) certified as malaria-free in 2016, while the remaining 28 countries were categorized as controlling malaria. Hereafter the term, eliminating countries, refers to the E2020 countries and Sri Lanka. Surveys were completed between 1 November 2017 and 19 November 2018. Overall, 91% of participating countries distributed LLINs, 31% implemented IRS and 41% practiced LSM at the time of the survey. For more details regarding the scale and scope of vector control and surveillance operations, see Burkot et al*.* [20].

Only 8% of NMCPs reported having sufficient capacity to implement vector surveillance. In contrast, 57%, 56% and 28% of NMCPs had the capacity to implement LLINs, IRS and LSM activities, respectively. When the capacity limitations were analysed by the country malaria status, the countries controlling malaria more frequently expressed limitations than countries that were eliminating malaria (χ^2^ = 47.77, df = 3, p < 0.0001). In other words, the respondents from the controlling countries, more frequently expressed the existence of NMCP capacity limitations than those in eliminating countries.

### Intervention capacity by programmatic inputs

**Fig. 2:**
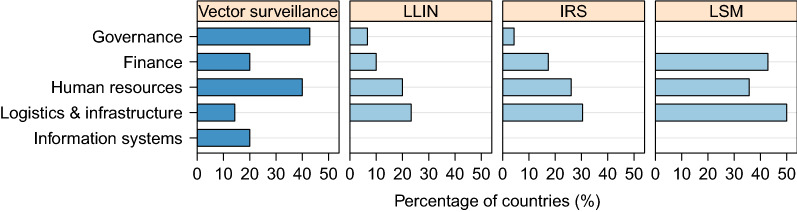
Relative comparison of the programmatic inputs (*y* axis) that limit ability of National Malaria Control Programmes to fully implement vector control activities (*x* axis). Here the bars represent the proportion of countries that indicated each input is limiting

Vector surveillance implementation by NMCPs was limited by governance (42%), human resources (40%), finance (20%), information systems (20%) and logistics and resources (14%). For vector control (LLINs, IRS and LSM), the proportional responses in these same categories differed with the majority of respondents highlighting limitations in logistics and resources (53%), followed by human resources (43%), funding (36%) and governance (10%) (χ^2^ = 21.48, df = 4, p = 0.0002; Fig. 2). Thus, governance (strategic planning) was much more limiting for vector surveillance activities than for other vector control deployment activities. Respondents did not identify information systems as a limitation for LLINs, IRS or LSM deployment.

**Fig. 3:**
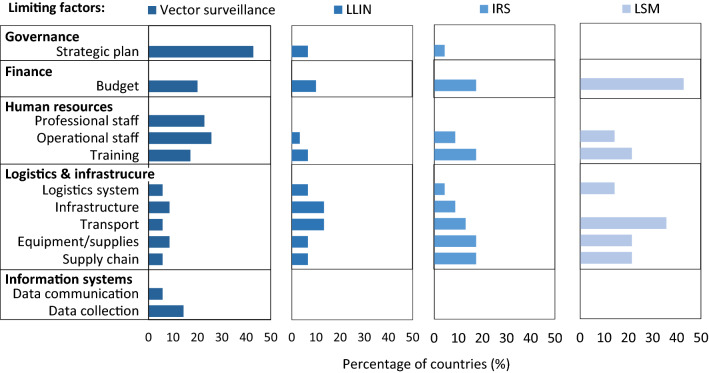
The subcategories of programme inputs limiting National Malaria Control Programmes to fully implement vector surveillance and vector control interventions. Percentages were calculated using the number of countries that deploy each intervention as the denominator and show the percentage of countries that reported a programme subcategory as limiting

The main subcategories of programmatic inputs that limited vector surveillance activities were strategic plans, operational staff, professional staff and the budget (Fig. 3). Many of the respondents specifically noted that the strategic plan was limited in the scale or scope of vector surveillance activities. While for LLINs, IRS and LSM, the main input limiting subcategories were budget, training, equipment and supplies, transport and operational staff (Fig. 3).

**Fig. 4:**
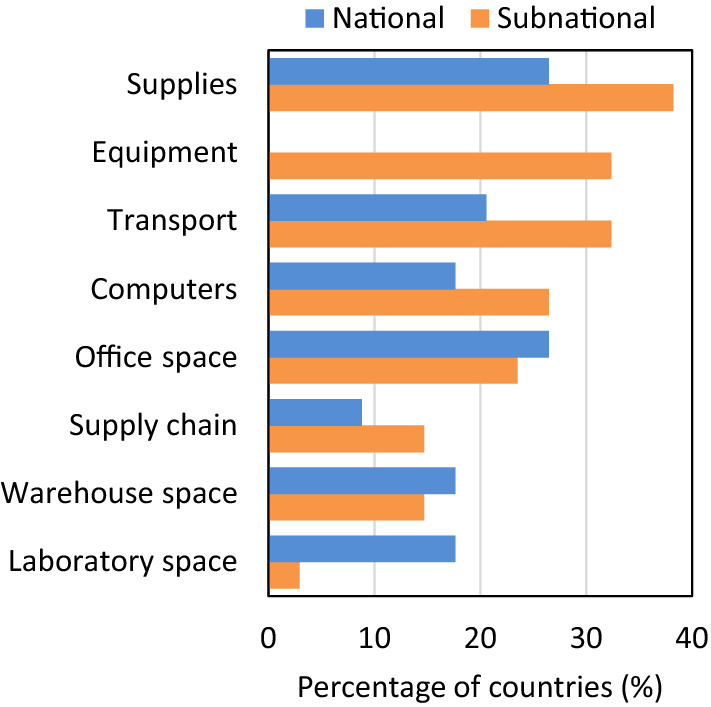
Components of logistics and infrastructure reported by countries survey participants as limiting National Malaria Control Programme implementation of vector surveillance at the national and subnational levels

**Fig. 5:**
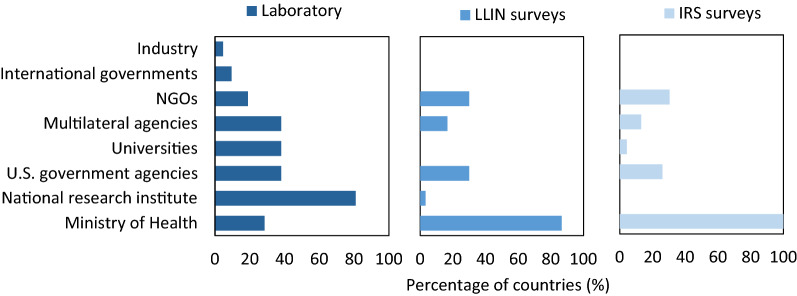
Partners supporting vector surveillance through direct assistance to laboratories, or surveys to monitor LLINs or IRS. Percentages were calculated using the denominator of the number of countries that had laboratory access or undertook LLINs or IRS activities

Resource limitations for vector control activities differed significantly at the national and subnational levels for both vector surveillance and the individual interventions used for control (χ^2^ = 20.21, df = 8, p = 0.009; Fig. 4). Subnational (i.e. provincial and district level) malaria control programmes more frequently had shortfalls in supplies, equipment, transport, computers and office space. At the national level, supplies, office space and transport were inadequate (Fig. 5).

NMCPs in 60% (n = 21) of countries had access to an entomology laboratory (e.g. molecular (PCR) or immunology (ELISA) capacity). All countries with ELISA capabilities (31% (n = 11)) also were PCR capable (42% (n = 15)). Insectaries were maintained in 57% (n = 18) of the countries in which colonies of *Anopheles arabiensis*, *Anopheles funestus *sensu stricto (*s.s.*), *Anopheles gambiae s.s.* and *Anopheles merus* in Africa; *Anopheles aconitus*, *Anopheles balabacensis*, *Anopheles dirus*, *Anopheles maculatus*, *Anopheles minimus*, *Anopheles sinensis* and *Anopheles sundaicus* in the Asia–Pacific; and *Anopheles albimanus* in the Americas were maintained. In addition, 31% (n = 11) of NMCPs had semi-field facilities.

Malaria control programmes collaborate with external partners or organizations such as universities, multilateral agencies or U.S. government agencies to varying degrees (Fig. 5). Partners supporting laboratories differed from those supporting surveys of intervention access and use (e.g., LLIN and IRS surveys) (χ^2^ = 60.39, df = 7, p < 0.0001) with entomological laboratories mainly supported by national research institutes (Fig. 5). LLIN and IRS use and coverage surveys were primarily conducted by the Ministry of Health (Fig. 5). External support for surveys in the Asia–Pacific was uncommon and significantly less than in Africa for LLINs (χ^2^ = 11.64, df = 4, p = 0.020), and almost significant for IRS (χ^2^ = 9.04, df = 4, p = 0.060).

### Staffing and training for capacity building

**Fig. 6:**
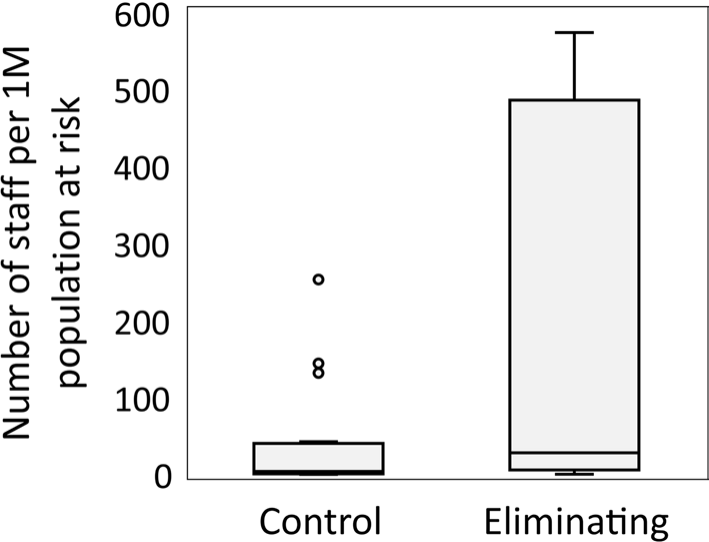
The median number of vector control entomology staff per national malaria control programme for countries eliminating malaria compared with those controlling malaria

**Fig. 7:**
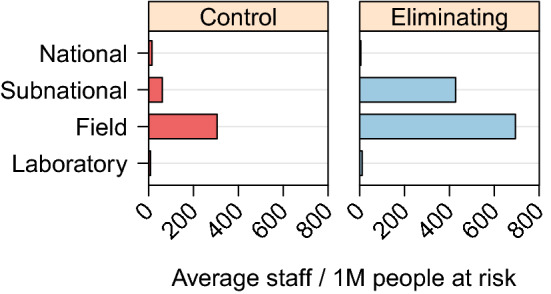
Relative composition of staffing across programmatic levels for national malaria control programmes that are controlling or eliminating malaria

Eliminating countries were better staffed compared with countries controlling malaria (β = -0.028, se = 0.011, p = 0.019). The median number of staff in eliminating programmes was 28 per 1 million people at risk, while control countries had a median of 4 staff per 1 million people at risk (Fig. 6). Within each programme, staff work at the national, subnational (provincial/district), field and laboratory postings, and the ratios of these staffing allocations was not significantly different between eliminating and control programmes (F_(1,31)_ = 0.397, p = 0.258; Fig. 7). However, as eliminating programmes had more staff, the number of field and provincial staff was also greater than in control programmes.

**Fig. 8:**
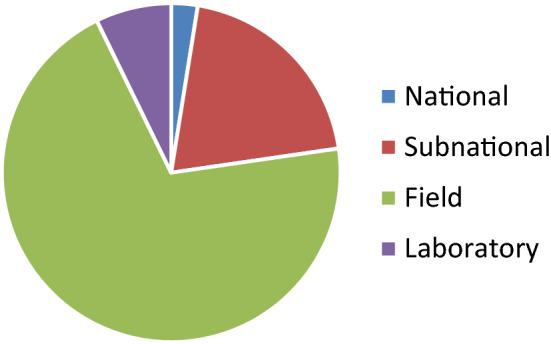
Proportional shortfalls in staffing capacities of national malaria control programmes

Reflecting the high resource requirements for elimination programmes, all eliminating country respondents opined that present staff numbers were not adequate to undertake all vector surveillance and control activities, while only 55% of control countries indicated that their programmes were under-staffed (χ^2^ = 3.62, df = 1, p = 0.057). There was no difference in the perceived relative need for additional staff for countries controlling or eliminating malaria (F_(1,31)_ = 1.24, p = 0.291) with both control and eliminating countries indicating a need to double the number of staff to attain sufficient staffing capacity. Overwhelmingly the greatest need for additional staff was at the subnational (provincial/district) and field positions (Fig. 8). Over half of control and eliminating country staff were engaged in both malaria and dengue control (62% for countries controlling malaria and 71% for eliminating countries).

More eliminating countries (86%) had an established system for staff training and capacity building compared to countries controlling malaria (50%) but this difference was not significant (χ^2^ = 1.604, df = 1, p = 0.205). The primary opportunities for training or capacity building was through ad hoc on the job training (11 countries), as well as regional or national vector control courses (10 countries). A limited number of countries had mechanisms for post-graduate training (n = 3), and 1 country had a structured training-of -trainers programme. Staff that participated in training were field entomologists and vector control officers (12 countries each) followed by programme managers (9 countries) and provincial entomologists (10 countries).

## Discussion

The capacity of NMCPs to implement vector surveillance activities was assessed against a conceptual framework to: (a) identify bottlenecks to implementing vector surveillance activities, (b) assess the quality of vector surveillance programmes against existing best practices [20], and (c) prioritize capacity bottlenecks to be addressed to improve the quality of vector surveillance programmes. The framework categorized programmatic elements (inputs) to enable the delivery of malaria programmes services, including vector surveillance. Governance, in the form of strategic planning, is a foundational input providing structure for the other inputs (finance, human resources, logistics/infrastructure, and information systems).

These inputs of NMCPs do not act independently and the successful implementation of vector surveillance activities depends on the capacity of all inputs. Thus, limitations in any programmatic input can limit operational activities which has a cascading impact on outputs (improved vector surveillance and informed decision-making), which, in turn, impacts vector control operations. This was particularly evident in the overall capacity of countries to implement vector surveillance compared with LLIN or IRS control implementation. Vector surveillance was most heavily limited by strategic planning, with many countries reporting that “*vector surveillance wasn’t seen as a priority*”. Not surprising, only a small fraction of countries (8%) reported having sufficient capacity to implement vector surveillance activities. In contrast, LLIN and IRS control activities are generally well integrated into malaria strategic plans and limitations were mostly in logistics and resources. While national malaria policies are strongly influenced by WHO recommendations, it is essential to also acknowledge the role of local politics [25] and the need to advocate for local champions to promote the importance of vector surveillance.

When implementing LLINs and IRS, human resources, logistics and infrastructure were perceived to be the primary limitations to programme success [26]. Management staff are essential for the success of malaria programmes [27] and this study found that management staff numbers only limited vector surveillance activities, not intervention deployment. In contrast, countries reported being hindered by a lack of operations staff and training for deployment of all interventions (vector surveillance, LLINs, IRS and LSM). This is an important delineation, because the implementation of vector surveillance hinges on the availability of higher degree educated staff (graduate/post-graduate) to plan as well as to interpret data for vector control decisions, while the implementation of vector control requires a large team of operational staff (technicians and auxiliary staff).

Elimination programmes require much higher numbers of operational/field staff [3]. Here, the median number of staff in eliminating programmes was 28 per 1 million people at risk, while control countries had a median of 4 staff per 1 million people at risk. Yet what was interesting was that controlling countries were less likely to indicate that their programmes were under-staffed. Which reflects a greater focus on vector control implementation compared with implementing a responsive evidence-based programme that includes entomological surveillance. In fact, as countries move towards elimination, there will be a need to recruit and train more operational staff to support the intensified activities including vector surveillance [28]. The respondents overwhelmingly identified that the greatest need for staffing and training was at the subnational and field positions, and this is supported by previous landscaping analyses (Burkot and Gilbert, pers. commun). It is unlikely that controlling countries will make significant progress unless huge investments also go towards building the numbers and capacity of programmatic staff.

One limitation in this study was the use of open text questions in a rapid assessment tool. While the intent was not to be prescriptive in coercing participants to select from a limited number of answers, and thus biasing responses, it may be that the respondents only reported their perceived greatest programme limitations. Consequently, some programme capacity limitations may have been under-reported by participants. Similarly, some inputs were not reported as limiting, such as information systems for vector control activities despite data collection on paper and management in excel being the most commonly reported “data information system” in this survey [20]. These apparently conflicting response suggests that data management systems are not perceived as an input that primarily hampers activities and/or that there is an under appreciation of the value of vector surveillance data in decision-making [20]. On the other hand, the use of an online survey tool facilitated rapid collection of a large quantity of capacity and capability information from numerous country programmes. While needs assessments traditionally collect quantitative information via a series of interviews, the utilization of an online tool that facilitated coding of the information into thematic areas was hugely beneficial to streamlining analysis [23] and essential in the wake of the COVID-19 pandemic.

The fundamental purpose of entomological surveillance is to inform programmatic decisions for effective vector control operations. Entomological data should be considered when stratifying transmission risk, planning vector control, responding to outbreaks, and assessing the impact of interventions [2]. However, significant gaps exist between the data collected, and the actual use of data in making programmatic decisions. For example, insecticide resistance phenotypes of adult vectors was measured, at least annually, in 75% of countries, and yet only 18% of countries considered this data when selecting insecticides for programmatic use [20]. Overall, this was likely a consequence of multiple factors interacting to limit vector surveillance programmes: a lack of political will [25], strategic support and professional staff and poor data management.

To enable proactive responses to potential and emerging threats to malaria control, NMCPs need updated vector surveillance plans and guidelines that facilitate evidence-based decision based on entomological surveillance. Full implementation of comprehensive surveillance plans then requires adequate financing and well-trained human resources at both the national and subnational levels with the infrastructure to collect and manage data to make enable evidence-based decisions to guide vector control strategy deployment stratified to maximize impact on malaria transmission.

## Conclusion

The Global Technical Strategy for Malaria 2016–2030 elevates malaria surveillance as a core intervention [4]. Strategic planning and a lack of well-trained entomologists most heavily limited vector surveillance. Eliminating countries generally had more operational/field staff compared to controlling countries, and also were more likely to have an established system for staff training and capacity building. It is unlikely that controlling countries will make significant progress unless huge investments also go towards building the numbers and capacity of programmatic staff. Significant gaps exist between the entomological data collected and the use of data, this was likely a consequence of multiple factors interacting to limit vector surveillance programmes: a lack of strategic support, a lack of professional staff and poor data management in addition to an under appreciation of the value of vector surveillance data. Undoubtedly, strong vector surveillance and control system supported by strong governance and well-trained staff will facilitate the drive towards malaria elimination.

## Data Availability

Aggregated data is available, but country specific data will remain confidential unless written authorization to release the data is granted by the specific national malaria control authorities. Aggregated data are available in the JCU Tropical Data Hub repository: https://doi.org/10.25903/5dba680ab310.

## Abbreviations

IRS: Indoor residual spraying
ITN: Insecticide-treated nets
LLIN: Long-lasting insecticidal net
LSM: Larval source management
NMCP: National Malaria Control Programme
PMI: President’s Malaria Initiative
WHO: World Health Organization
